# Soil Nitrogen Availability and Plant Genotype Modify the Nutrition Strategies of *M. truncatula* and the Associated Rhizosphere Microbial Communities

**DOI:** 10.1371/journal.pone.0047096

**Published:** 2012-10-15

**Authors:** Anouk Zancarini, Christophe Mougel, Anne-Sophie Voisin, Marion Prudent, Christophe Salon, Nathalie Munier-Jolain

**Affiliations:** INRA, UMR1347 Agroécologie, Dijon, France; University College London, United Kingdom

## Abstract

Plant and soil types are usually considered as the two main drivers of the rhizosphere microbial communities. The aim of this work was to study the effect of both N availability and plant genotype on the plant associated rhizosphere microbial communities, in relation to the nutritional strategies of the plant-microbe interactions, for six contrasted *Medicago truncatula* genotypes. The plants were provided with two different nutrient solutions varying in their nitrate concentrations (0 mM and 10 mM). First, the influence of both nitrogen availability and *Medicago truncatula* genotype on the genetic structure of the soil bacterial and fungal communities was determined by DNA fingerprint using Automated Ribosomal Intergenic Spacer Analysis (ARISA). Secondly, the different nutritional strategies of the plant-microbe interactions were evaluated using an ecophysiological framework. We observed that nitrogen availability affected rhizosphere bacterial communities only in presence of the plant. Furthermore, we showed that the influence of nitrogen availability on rhizosphere bacterial communities was dependent on the different genotypes of *Medicago truncatula*. Finally, the nutritional strategies of the plant varied greatly in response to a modification of nitrogen availability. A new conceptual framework was thus developed to study plant-microbe interactions. This framework led to the identification of three contrasted structural and functional adaptive responses of plant-microbe interactions to nitrogen availability.

## Introduction

In the major agricultural areas of Europe, cropping systems are still based on a large use of inputs (pesticides, nitrogen fertilizers, energy and water) and have poor environmental performances. However, agricultural production faces the challenge of supplying an increasing world population without hampering ecosystems [1]. In this context of highly pressured agricultural production using low levels of chemical fertilisers, cropping strategies could take advantage of existing plant-microbe interactions that can improve both plant growth and yield [2]–[4].

In the rhizosphere, microbial communities are able to interact with plants and, for some type of interactions, improve plant growth and health [5]–[10]. In particular, three main types of interactions are able to increase nutrient availability for plants. The first one is a symbiotic interaction between the roots of leguminous plants and N_2_-fixing bacteria such as *Rhizobium*, which convert atmospheric N_2_ into ammonia in specific organs called nodules [11]. A second type of interaction occurs between the roots of most terrestrial flowering plants and arbuscular mycorrhizal fungi which are able to absorb and translocate phosphate from the soil to the plant [12]. Finally, the third type of interactions includes a large diversity of soil microbes involved in the mineralisation of soil organic matter and produce mineral nitrogen from organic nitrogen. For instance, soil-nitrifying bacteria can produce nitrate that enhances plant growth [13]. Legume plants are able to benefit from those three types of interactions. Indeed, they can establish symbiotic interactions with N_2_-fixing bacteria, arbuscular mycorrhizal fungi, soil-nitrifying bacteria and other rhizosphere microbes for their nutrition. Among legumes, *Medicago truncatula* is an interesting model to study plant-microbe interactions, as it is commonly used in genetical, ecophysiological and microbiological studies [14]–[16].

Interactions between the plant and the rhizosphere microbial communities are bidirectional and rhizodeposition is considered to be the basis of these interactions through the release of organic compounds from the plant roots [17]–[20]. On average, 17% of the net C fixed by photosynthesis is lost by roots through rhizosphere respiration (12%) and rhizodeposition (5%) [20]. Some compounds identified in root exudates play an important role as signalling molecules in root-microbe interactions such as flavonoids. These molecules are present in the root exudates of legumes and they are known to activate *Rhizobium Nod* genes responsible for nodulation processes [21], [22]. Above all, rhizodeposition is an input of organic carbon into the soil, where it is directly available for microbial community growth.

Therefore, even if plant-microbe interactions can facilitate nutrient uptake by the roots, it also implies carbon cost for the plant due to C losses by rhizodeposition. In order to unravel the C and N fluxes in the plant, a conceptual ecophysiological framework has been developed to decompose the physiological bases involved in *Medicago truncatula* C and N nutrition [15], [23]. However, while plant-microbe interactions can have drastic effect on the plant C and N nutrition, these previous studies did not consider the plant interactions with their associated rhizosphere microbial communities. Therefore, this *Medicago truncatula* ecophysiological framework needs to be extended to plant-microbe interactions.

Moreover, plant-microbe interactions are likely to be affected by the soil quality, such as N availability. Firstly, this N effect could be due to direct effect of nutrient availability on soil microbial communities [24] or on plant nitrogen status [25] which could also affect root exudation and microbial colonisation [26]. Secondly, this nutrient effect could be due to a decreased plant dependence on plant-microbe interactions as N being readily available to the roots. Indeed, it has been widely reported that soil mineral N inhibits legume nodulation as it costs less energy for legumes to take up N from soil than to fix N through symbiotic interaction with *Rhizobium* [27]–[29]. Finally, we showed in a previous study that the rhizosphere bacterial communities were affected by the plant genotypes which could be discriminated by their contrasted C and N nutritional strategies [30].

The aim of this work was therefore to study the effect of both N availability and plant genotype on the plant associated rhizosphere microbial communities, in relation to the nutritional strategies of the plant-microbe interactions for six contrasted *Medicago truncatula* genotypes.

For that purpose, a greenhouse experiment was conducted on six genotypes of *Medicago truncatula* selected for their genetic diversity [31]. These plants were grown on soil that hosts many symbiotic microbes [32]. The plants were provided with two different nutrient solutions varying in their nitrate concentrations (0 mM and 10 mM). First, the influence of both N availability and *Medicago truncatula* genotype on the genetic structure of the soil bacterial and fungal communities was determined. Secondly, the different nutritional strategies depending on both N availability and plant genotype were evaluated using an ecophysiological framework.

We observed that N availability affected rhizosphere bacterial communities only in presence of the plant. Furthermore, we showed that the impact of N availability on rhizosphere bacterial communities was dependent on the *Medicago truncatula* genotype with contrasted nutritional strategies in response to a modification of N availability.

## Materials and Methods

### Plant material and culture conditions

Six *Medicago truncatula* genotypes (DZA315-16, DZA315-26, F83005-5, Jemalong-A17, Jemalong-J6 and SA028064) were used in this study, chosen for their contrasted genetic diversity [31]. The seeds of *Medicago truncatula* genotypes were scarified and surface-sterilized [16], vernalized at 4°C during 48 h and germinated on 0.7% (w/v) water agar plates at 25°C in the dark. A single germinated seed of each *Medicago truncatula* genotype was sown in a pot containing a silt-clay loam soil (Mas d'Imbert, France) which hosts many symbiotic microbes [32]. This soil is characterized by low mineral nitrogen (N) content: 0.018 g.kg^−1^ NO_3_ and 0.002 g.kg^−1^ of NH_4_. The physicochemical characteristics of this soil were determined as: 11% sand, 51% silt, 38% clay, pH = 8, 14.5 g.kg^−1^ of organic carbon, 1 g.kg^−1^ of N and 1 g.kg^−1^ of P_2_O_5_.

The plants were cultivated up to the end of their vegetative period at a density of 45 plants.m^−2^ in a greenhouse, under a photoperiod of 15 h and a temperature of 26/21°C (day/night). Supplementary artificial light was supplied with sodium lamps (MACS 400 W; Mazda, Dijon, France) to complement photosynthetically active radiation. The plants were watered with two different nutrient solutions varying in their nitrate concentration (0 mM and 10 mM) [15]. An automatic watering was used to maintain soil moisture at 45% of its maximum soil water-holding capacity.

### Genetic structure of the soil's bacterial and fungal communities

#### Extraction and purification of total DNA from soil sample

DNA was extracted from two different compartments: bulk soil and rhizosphere soil as previously described by Mougel et al. [16]. The bulk soil was the soil contained in control pots with no plants but kept in the same environmental conditions as the others. The rhizosphere soil was obtained after manually separating the root system with adhering soil from the container and by washing the root system under agitation (vortex at 30 hertz for 1 minute) in 50 ml of sterile 0.025 M K_2_SO_4_ solution. The root system was discarded and the soil was collected after centrifugation at 9000 g for 10 min. For bulk soil, four replicates were used per N treatment, and for the rhizosphere soil compartment four replicates per genotype and N treatment were used. Each sample was weighed, frozen in liquid nitrogen and conserved at −80°C. The DNA extraction procedure was based on chemical and mechanical extraction as previously described by Ranjard et al. [33] and by Mougel et al. [16]. DNA preparations were quantified as previously described by Mougel et al. [16].

#### Automated RISA fingerprinting

The bacterial ribosomal IGS were amplified with primers S-D-Bact-1522-b-S-20 (3′ end of 16S genes) and L-D-Bact-132-a-A-18 (5′ end of 23S genes) [34] for bacterial automated ribosomal intergenic spacer analysis (B-ARISA). The fungal ITS1-5,8S-ITS2 region was amplified with the primers ITS1-F (3′ end of 18S genes) [35] and 3126T (5′ end of 28S genes) [36] for fungal automated ribosomal intergenic spacer analysis (F-ARISA). PCR conditions, PCR template preparation for DNA sequencer loading and electrophoresis conditions were described by Ranjard et al. [33].

### Symbiotic microbial infections

The intensity of nodulation was evaluated using a visual scale taking into account nodulation intensity, size and functionality [25] on the four root systems per genotype and per N treatment used for the characterization of the genetic structure of microbial communities. The scale includes five scores: 0 (absence of nodules), 1 (only some white small size nodules), 2 (both white and pink small size nodules), 3 (mainly pink larger size nodules), and 4 (many pink large size nodules).

The efficiency of the arbuscular mycorrhizal (AM) infection was determined on five root systems per genotype and per N treatment. A non-vital staining procedure was used to allow visualization of total AM fungi [37]. The intensity of root cortex colonisation by AM fungi was determined as previously described [38] using MYCOCALC software http://www.dijon.inra.fr/mychintec/Mycocalc-prg/download.html) and expressed as: frequency (*F*%) and intensity (*M*%) of colonisation of the root cortex; and arbuscular abundance of the root cortex (*A*%).

### Structural and functional descriptors of plant-microbe interactions

#### Plant growth

At three sampling dates (340, 634 and 934 degree-days after sowing), representing three vegetative developmental stages (4 leaves, first ramification and second ramification) [16], [39], four plants per genotype were harvested and the leaf area was measured (LI-3100 Area Meter, Li-Cor Inc., Lincoln, NE, USA); shoot and root biomass and total nitrogen (N) content were determined after drying at 80°C during 48 h. The data obtained for these three plant harvests were used to calculate integrative ecophysiological framework parameters.

#### C and N nutritional strategies using an ecophysiological framework

The various plant genotypes could employ contrasted carbon (C) and nitrogen (N) nutritional strategies to reach similar growth among N treatments. An ecophysiological framework [15], [23] was used to study these different C and N nutritional strategies. This framework linked four integrative variables (leaf area, total biomass, below-ground biomass and total amount of N in plant) with four intermediate parameters: the radiation use efficiency (RUE) for biomass production, the root∶total biomass ratio (RTR), the plant-specific N uptake (SNU) and the conversion factor of N to leaf area (NLA) [15], [23].

#### Rhizodeposition

The carbon (C) rhizodeposition was quantified with ^13^C content in the rhizosphere soil at 934 degree-days after sowing. The labelling experiment was conducted in a gas-proof chamber as in Jeudy et al. [40]. The environmental parameters of this chamber were: a 14/10 h day/night photoperiod, a temperature of 22°C, 60% relative-humidity and a photosynthetically active radiation of 650 µmol photons.m^−2^.s^−1^ (108 DULUX L 55 W lamps, APPRO5-21850, Saint Appolinaire, France). The atmospheric CO_2_ concentration within the labelling chamber was measured and maintained at 380 µL L^−1^ CO_2_ [40]. The plants were exposed for 10 h to a ^13^C-enriched atmosphere (63.8% ^13^C/^12^C). Isotopic compositions of gases in the labelling chamber were controlled by a modular gas analyser (SIC MAHIAC S710, Hamburg, Germany) using non dispersive infrared. After two days of chase, the rhizosphere soil was obtained after manually separating the root system with adhering soil from the container and by washing the root system under agitation (vortex at 30 hertz during 1 minute) in 50 ml of sterile 0.025 M K_2_SO_4_ solution. Each sample was frozen in liquid nitrogen, conserved at −80°C and freeze-dried for further ^13^C analysis using a continuous-flow isotope ratio mass spectrometer (Sercon, Crewe, UK) coupled to a C-N elemental analyser (Thermo Electron NC2500, Courtaboeuf, France). The quantities of carbon (QC) in the rhizosphere soil acquired during the labelling period were calculated as follows:
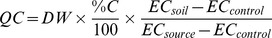
(1)where DW is the soil dry weight, %C the percentage of carbon in the soil dry weight (w/w), EC_soil_ and EC_control_ are respectively the ^13^C abundance in the rhizosphere soil of the labelled plant or the non-labelled control plant and EC_source_ is the ^13^C enrichment of the atmosphere.

#### Nitrogen nutrition index

A nitrogen nutrition index (NNI) was calculated in order to estimate the plant N nutrition level. The N nutrition level was considered as optimal when the NNI was close to 1 and a N-deficiency or a N-excess nutrition level was revealed by respectively a NNI lower or higher than 1. The NNI was the ratio between shoot N concentration and the “critical shoot N concentration” (%Nc) which was defined by the critical dilution curve, as follows:
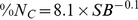
(2)where SB was shoot biomass (g) [25], [41].

### Statistical analysis

All statistical analyses were performed using the statistical software package R 2.9.2 (R Development Core Team, 2009, Vienna, Austria). Only differences significant at P<0.05 were considered.

Nodulation intensity, mycorrhization parameters, biomass, leaf area, total amount of nitrogen (N), rhizodeposition and N nutrition index were analysed using 2-factor ANOVA tests.

Radiation use efficiency (RUE), root∶total biomass ratio (RTR), specific N uptake (SNU) and N to leaf area ratio (NLA) were analysed using 2-factor ANCOVA test.

The principal component analysis (PCA) describing the genetic structure of bacterial and fungal communities of the different *Medicago truncatula* genotypes in contrasted soil N availability was performed as reported by Ranjard et al. [33]. PCA describing the genetic structure of bacterial and fungal communities and Monte Carlo tests were performed using the Ade4TkGUI software [42].

The genotype, soil N availability and interaction between genotype and soil N availability effects were tested on the first four PCA components that described the genetic structure of bacterial communities. We calculated the different sample coordinates on the first four PCA components using the R software.

## Results

### Soil N availability modified the genetic structure of soil bacterial communities only in presence of the plant

Soil N availability effect on the genetic structure of soil microbial communities was assessed for six *Medicago truncatula* genotypes considering the entire soil microbial communities, both bacterial and fungal. Moreover, in order to estimate to what extent the plant affects bacterial and fungal communities in different N availability contexts, analyses were carried out in two soil compartments (bulk soil and rhizosphere soil).

No matter the soil compartment, no significant effect of soil N availability was observed on the fungal communities (Table 1). In contrast, for bacterial communities, a significant effect of N availability was observed in the rhizosphere soil but not in the bulk soil (Table 1). This indicates that N availability modified soil bacterial communities only in presence of the plant.

**Table 1:** Statistical analysis results of the effect of N treatment on both bacterial and fungal communities in the bulk soil and in the rhizosphere soil of different *Medicago truncatula* genotypes.

	Bacterial communities	Fungal communities
Bulk soil	0.09^ns^	0.91^ns^
Rhizosphere soil	0.0002***	0.32^ns^
DZA 315-16	0.03*	0.19^ns^
DZA 315-26	0.03**	0.52^ns^
F 83005-5	0.03*	0.28^ns^
SA 028064	0.05*	0.07^ns^
Jemalong A17	0.14^ns^	0.43^ns^
Jemalong J6	0.45^ns^	0.31^ns^

Significance of ANOVA: ns, *, ** and *** indicate not significant and significant levels of 0.05, 0.01 and 0.001 respectively.

### Both N availability and *Medicago truncatula* genotype affected the genetic structure of rhizosphere bacterial communities

**Figure 1:**
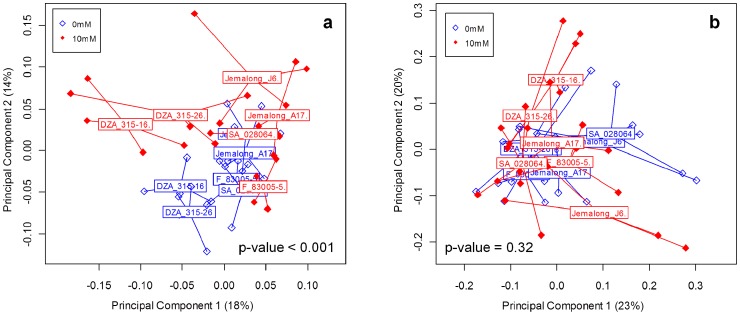
Genetic structure of bacterial and fungal communities associated to different genotypes of *Medicago truncatula* under contrasted soil N availability. Genetic structure of bacterial (**a**) and fungal (**b**) communities of different genotypes of *Medicago truncatula* (DZA315-16, DZA315-26, F8305-5, Jemalong-A17, Jemalong-J6 and SA028064) under contrasted soil N availability (0 mM and 10 mM N) was compared by Principal Component Analysis (PCA) at 934 degree-days after sowing.

To further assess both N availability and *Medicago truncatula* genotype effect on rhizosphere bacterial and fungal communities, principal component analysis (PCA) were performed (Fig. 1). The results show that the genetic structure of the rhizosphere bacterial communities was affected by both soil N availability and *Medicago truncatula* genotype (p-value<0.001) (Figure 1a). The first component representing 18% of the total variance was affected by *Medicago truncatula* genotype (Table 2). Indeed, DZA315-16 and DZA315-26 were separated from the other genotypes on the first axis (Figure 1a). The second component represented 14% of the total variance was affected by the *Medicago truncatula* genotype, the soil N availability and the interaction (Table 2).

**Table 2:** Summary of statistical analysis (2-factor ANOVA) comparing the effect of genotype and N availability on the genetic structure of the rhizosphere bacterial communities.

	Principal Component 1(18%)¤	Principal Component 2(14%)¤	Principal Component 3(10%)¤	Principal Component 4(8%)¤
Genotype	5. 10^−7^***	2. 10^−4^***	0.48^ns^	0.03*
N treatment	0.86^ns^	8. 10^−7^***	0.009**	0.005**
Interaction	0.19^ns^	0.03*	0.22^ns^	0.05^ns^

¤ Represent the percentages of the total variability explained by the four first principal components in the PCA.

Significance of ANOVA: ns, *, ** and *** indicate not significant and significant levels of 0.05, 0.01 and 0.001 respectively.

Moreover, while the rhizosphere bacterial communities of DZA315-16, DZA315-26, F83005-5 and SA028064 were affected by soil N availability, those of Jemalong-A17 and Jemalong-J6 were not (Table 1). Therefore, N availability effect on rhizosphere bacterial communities was dependent on the plant genotype. However the genetic structure of the rhizosphere fungal communities was neither affected by soil N availability nor by the plant genotype (p-value = 0.32) (Figure 1b).

As a result, unlike the genetic structure of the rhizosphere fungal communities, the genetic structure of the rhizosphere bacterial communities was contrasted among *Medicago truncatula* genotypes and soil N availability. Independent of N availability, genotypes DZA315-16 and DZA315-26 differed from the four other genotypes for their rhizosphere bacterial communities. Moreover, the soil N availability affected differently the rhizosphere bacterial communities according to the *Medicago truncatula* genotype considered.

### N availability sharply affected nodulation intensity but only slightly affected arbuscular mycorrhizal colonisation

*Medicago truncatula* forms two major symbioses with specific bacterial and fungal communities (Rhizobia and Arbuscular mycorrhiza, respectively). These interactions are both known to have a positive feedback effect on plant nutrition. As such, particular attention was given to the response of these two major symbioses in the different experimental conditions.

**Figure 2:**
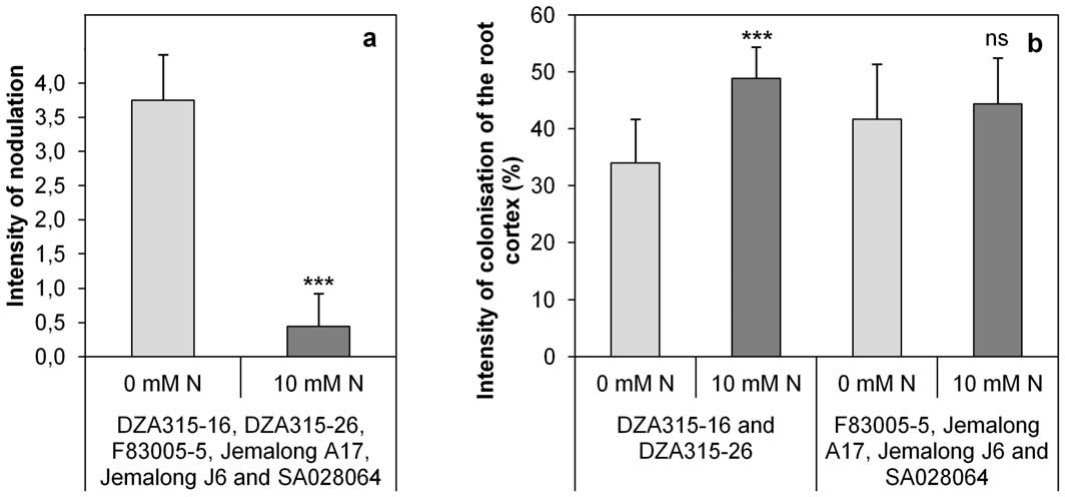
Contrasted symbiotic microbial infection among different *Medicago truncatula* genotypes and soil N availability. **a.** Intensity of nodulation of the six *Medicago truncatula* genotypes studied under contrasted soil N availability (0 mM and 10 mM N). The intensity of nodulation was evaluated using a visual scale [25] which includes five scores: 0 (absence of nodules), 1 (only some white small size nodules), 2 (both white and pink small size nodules), 3 (mainly pink larger size nodules), and 4 (many pink large size nodules). **b.** Intensity of colonisation of the root cortex (M%) under contrasted soil N availability (0 mM and 10 mM N) for the group of genotype DZA315-16/DZA315-26 and the other genotypes.

The results show that the rhizobia symbiotic association with *Medicago truncatula* was affected by the soil N availability but not by the plant genotype (Table 3). Actually, without N addition, the nodules of each genotype seemed functional while under high N availability (10 mM N), all the genotypes had either no nodules or only a few small white ones (Figure 2a).

**Table 3:** Summary of statistical analysis (2- factor ANOVA) comparing the effect of genotype and N availability on the symbiotic microbial infection.

	Intensity of nodulation	Mycorrhization parameters		
		F%	M%	A%
Genotype	0.06^ns^	0.88^ns^	0.53^ns^	0.36^ns^
N treatment	2. 10^−6^***	0.92^ns^	0.002**	0.41^ns^
Interaction	0.29^ns^	0.10^ns^	0.18^ns^	0.24^ns^

Significance of ANOVA: ns, ** and *** indicate not significant and significant levels of 0.01 and 0.001 respectively.

The efficiency of mycorrhizal colonisation was assessed through three measured parameters: frequency (F%) and intensity of root cortex colonisation (M%) and arbuscular abundance of the mycorrhized root cortex (A%). Neither soil N availability nor genotype had significant effect on the frequency of root cortex colonisation (F%) and arbuscular abundance of the mycorrhized root cortex (A%) (Table 3). However, the root cortex colonisation intensity (M%) was affected by the soil N availability (Table 3). Indeed, the root cortex colonisation intensity (M%) of genotypes DZA315-16 and DZA315-26 was affected by N availability (Figure 2b). Under high N availability (10 mM N) these two genotypes had significantly higher root cortex colonisation intensity than without N addition (0 mM N) (Figure 2b). Thus, N availability led to structural modifications in the arbuscular mycorrhizal infection of the two genotypes DZA315-16 and DZA315-26.

### N availability differentially affected the nutritional strategies of the plant according to *Medicago truncatula* genotype

In order to get a better insight into the putative contrasted C and N nutritional strategies of the interaction between *Medicago truncatula* and their rhizosphere bacterial communities we measured the effect of the different factors on the plant growth. The effect of the soil N availability, the plant genotype and the interaction between soil N availability and plant genotype were estimated based on several measured or estimated ecophysiological traits (Table 4). Our experiments show that specific N uptake was affected by the plant genotype (Table 4). Also, the N nutritional index, the total amount of N, the specific N uptake and the N to leaf area conversion efficiency were affected by the soil N availability (Table 4). Thus, the plant N metabolism was affected by both the *Medicago truncatula* genotype and the N availability. However, the interaction of the plant genotype and the soil N availability did not affect the C and N nutritional strategies (Table 4).

**Table 4:** Summary of statistical analysis (2- factor ANOVA and ANCOVA) comparing the effect of genotype and N availability on the ecophysiological variables.

	Leaf area	Total biomass	Root∶total biomass ratio	RUE	Rhizodeposition	NNI	Total amount of N	SNU	NLA
Genotype	0.17^ns^	0.14^ns^	0.37^ns^	0.17^ns^	0.87^ns^	0.26^ns^	0.15^ns^	0.23*	0.12^ns^
N treatment	0.79^ns^	0.41^ns^	0.19^ns^	0.97^ns^	0.12^ns^	1. 10^−12^***	9. 10^−4^***	5. 10^−6^***	3. 10^−5^***
Interaction	0.65^ns^	0.93^ns^	0.51^ns^	0.58^ns^	0.20^ns^	0.96^ns^	0.66^ns^	0.24^ns^	0.85^ns^

Leaf area (cm^2^), total biomass (g), root∶total biomass ratio, radiation use efficiency (RUE) (g of biomass MJ^−1^ of PAR intercepted), rhizodeposition (µ C s^−1^ g^−1^ of root), nitrogen nutritional index (NNI), total amount of N (mg plant^−1^), specific nitrogen uptake (SNU) (g of N g^−1^ of root biomass) and conversion factor of nitrogen to leaf area (NLA) (cm^2^ of leaves g^−1^ of N).

Significance of ANOVA: ns, *, ** and *** indicate not significant and significant levels of 0.05, and 0.01 and 0.001 respectively.

**Figure 3:**
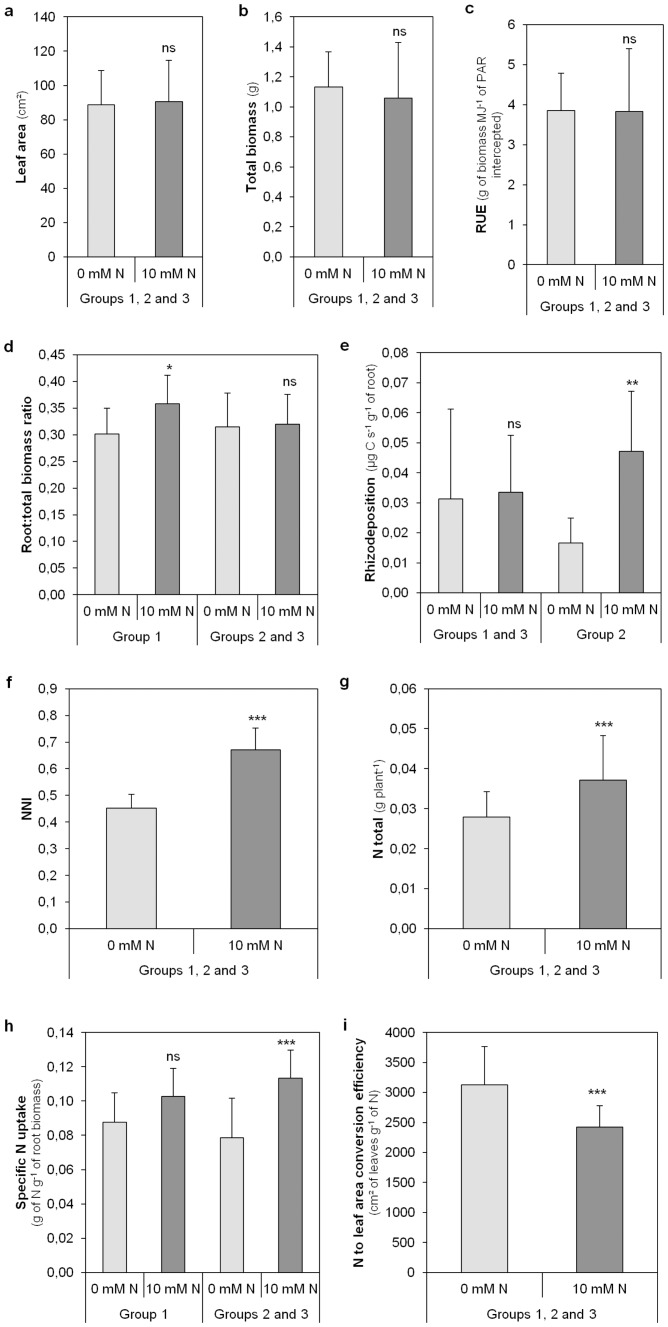
Modification of the C and N nutritional strategies among *Medicago trunctula* genotypes under different soil N availability. Leaf area (**a**), total biomass (**b**), radiation use efficiency (RUE) (**c**), root∶total biomass ratio (**d**), rhizodeposition (**e**), nitrogen nutritional index (NNI) (**f**), total amount of N (**g**), specific nitrogen uptake (SNU) (h) and conversion factor of nitrogen to leaf area (NLA) (**i**) of the three different groups of *Medicago truncatula* genotypes under contrasted soil N availability (0 mM and 10 mM N). Group 1 includes DZA315-16 and DZA315-26; Group 2 includes F83005-5 and SA028064; Group 3 includes Jemalong-A17, Jemalong-J6.

While all the *Medicago truncatula* genotypes decreased their N to leaf area conversion efficiency when the soil N availability increased (Figure 3i), the different genotypes responded differently. According to their response to N availability, the genotypes were classified into three main groups. The first group that included two genotypes (DZA315-16 and DZA315-26) was characterized by a significant increase of the root∶total biomass ratio following N availability (Figure 3d) and also by stable rhizodeposition (Figure 3e) and stable specific N uptake (Figure 3h) whatever N availability. A second and a third group of genotypes were identified, each comprising of two genotypes (F83005-5/SA028064 and Jemalong A17/Jemalong J6 respectively). These two groups were both characterized by a higher specific N uptake (Figure 3h) associated with stable root∶total biomass ratio whatever N availability (Figure 3d). F83005-5 and SA028064 were differentiated by a higher rhizodeposition process under high N availability (Figure 3e).

Thus, the nutritional strategies of *Medicago truncatula* changed with N availability in a genotype-dependent manner.

## Discussion

Plant and soil types are usually considered as the two main drivers of microbial community structure [6], [43], [44]. However, within a same soil type, nitrogen (N) availability is known to cause changes to the biomass, structure and activity of the soil and rhizosphere microbial communities [24], [45]–[47]. In our study, bacterial communities were affected by N availability only when these communities were interacting with a plant (Table 1). This finding supports the hypothesis suggested by Marschner and co-workers [46] that the effect of N availability on microbial communities was at least partly plant-mediated. In addition, plant nutritional strategies are also affected by both plant genotype and N availability [15]. To our knowledge, this is the first study to evaluate both N availability and plant genotype effect on both rhizosphere microbial communities and the nutritional strategy of the plant.

### A new conceptual framework to study plant-microbe interactions

**Figure 4:**
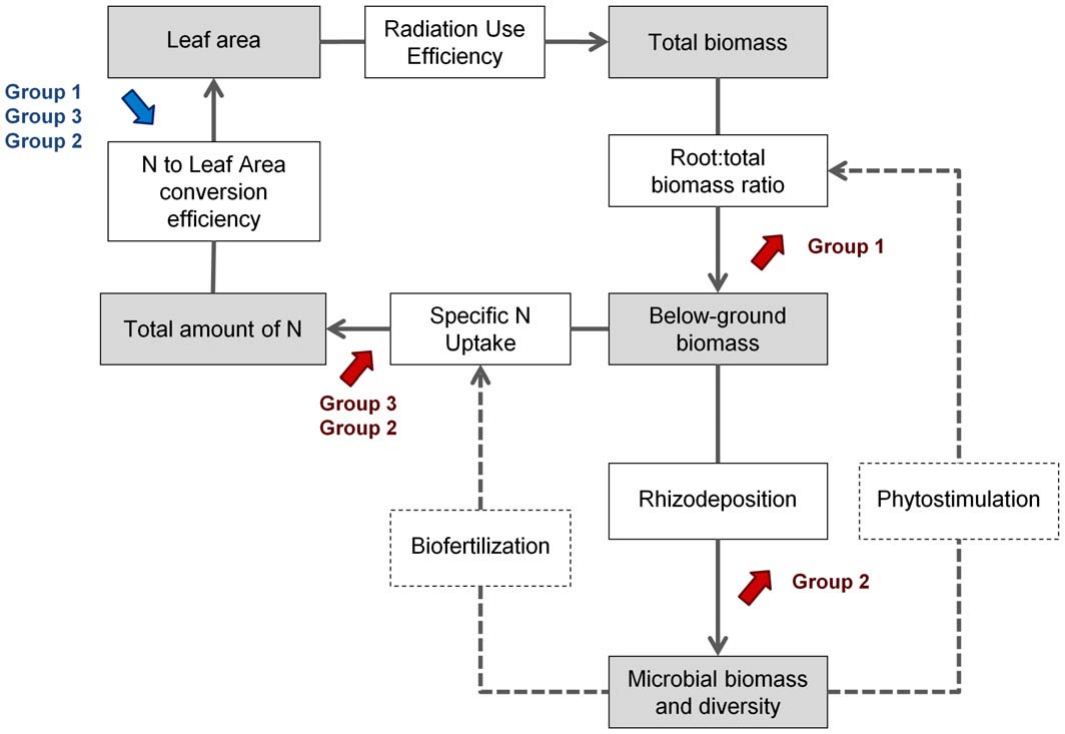
Contrasted adaptive responses of different *Medicago truncatula* genotypes to nitrogen availability. Contrasted adaptive responses of different *Medicago truncatula* groups of genotypes to nitrogen availability was assessed using a conceptual framework of plant functioning [15] extended to plant-microbe interactions. Group 1 includes DZA315-16 and DZA315-26; Group 2 includes F83005-5 and SA028064; Group 3 includes Jemalong-A17, Jemalong-J6. State integrative variables are in grey boxes, efficiency variables are in white boxes and non-measured putative effects are in dotted boxes. The red and blue arrows represent respectively a significant increase and decrease, from low to high N availability.

In microbial ecology, conceptual frameworks or models are used to analyse plant-microbe interactions but plant phenotype is currently viewed as a “black box” [48]–[51]. Classically measured plant traits (such as plant biomass or grain yield) result from the integration of a large number of plant physiological processes under the control of genetic and environmental factors. Conceptual ecophysiological frameworks based on the decomposition of complex plant traits into physiological processes are used to assess the phenotype and nutritional strategy at the whole plant level [52], [53]. To go further in our understanding of how the microbial communities are shaped *via* the selective effect in relation to their interactions with the plant under contrasted N availability, we extended a conceptual ecophysiological framework of plant functioning, previously developed for *Medicago truncatula* [15], to plant-microbe interactions (Fig. 4). The basic conceptual framework links four “state variables” characterizing plant growth (leaf area, total biomass, root biomass and the amount of plant N) to four “efficiency variables” representing the capacity of the plant to extract and utilize C and N resources (radiation use efficiency, root∶total biomass ratio, specific N uptake and N to leaf area conversion efficiency).

In order to extend this ecophysiological framework to plant-microbe interactions, we first added rhizodeposition, which is considered as the basis of plant-microbe interactions [17]–[20]. Then, we also considered the feedback effect of microbial communities on plant growth through two different ways. A beneficial effect of rhizosphere microbial communities on the plant could be directly related to a change in nutrient uptake which is defined by both exploration efficiency (structural modification) and specific N uptake (functional modification) [41]. The structural modification of root architecture could occur through phytostimulation processes with a regulation of the plant production of hormone such as auxins, cytokinins and gibberellins; and some microbial communities, such as N_2_-fixing bacteria and nitrifying bacteria, could supply the plant with nutrients through biofertilization processes [3], [10], [12], [54]–[56]. Thus, the extended conceptual framework integrates both plant effect on microbial communities through rhizodeposition and microbial community feedback through phytostimulation and biofertilization (Fig. 4).

### Identification of three different adaptive responses of plant-microbe interactions to N availability

Three different adaptive responses of plant-microbe interactions to N availability were identified and classified into three groups. These three groups presented both contrasted nutritional strategies and different genetic structures of microbial communities in response to N availability. The first group, comprised of DZA 315-16 and DZA 315-26, differed from the others in its response to N availability both, in its nutrition strategies and in the genetic structure of its rhizosphere microbial communities (Figures 1,3). Group 1 was characterized by a structural response to N availability, as these genotypes allocated more biomass to their root part without increasing their specific N uptake when N availability increased. Moreover, arbuscular mycorrhizal fungi in symbiosis with these plants also increased their exchange surface areas when N availability increased. The plant is known to display considerable developmental plasticity through root system architecture modulation in response to variations in the concentration and distribution of external N. This developmental plasticity could be due to a direct effect of N availability on the plant (autonomous pathway) or to an indirect effect via associated microbial communities (association pathway) [54]. Microbial communities, such as plant growth promoting rhizobacteria (PGPR) and arbuscular mycorrhizal fungi, can modify the root system architecture [9], [56]–[59]. While the plant and the microbial community effects could not be distinguished in our study, the structural response of DZA 315-16 and DZA 315-26 to N availability probably occurs through a phytostimulation process [10], [54]. Nevertheless, in our study, this structural response of the plant-microbe interaction to N availability did not allow an increase of N uptake by the plant. As plant biomass tended to decrease, this root developmental response must have had a high carbon cost for the plant that limited N uptake [41], [60]. If this structural response did not confer a benefit to the plant in pot conditions, the increase of root exploration could present an advantage in field conditions, wherein the increase of root prospection can permit access to a greater amount of soil and therefore nutrients.

The second and the third groups were characterized by a functional response to N availability increasing their specific N uptake without allocating more biomass to their root part (Figure 4). The increase of specific N uptake could be related either to a better capacity of the plant to acquire N by regulating for instance nitrate transporters or to an increase of N availability due to the soil mineralisers [10], [54], [55]. The second group, which included the F83005-5 and SA028064 genotypes, increased both specific N uptake and rhizodeposition processes. This group also modified the genetic structure of bacterial communities when passing from low to high N availability conditions. Nevertheless, these functional responses to the increase of N availability did not trigger a significant increase of the plant total N amount. As for group 1, the setting up of the plant-microbe interaction may have had a high carbon cost for the plant that limited plant growth and N uptake but to a lesser extent than for group 1.

The third group, comprising the Jemalong-A17 and Jemalong-J6 genotypes, modified neither rhizodeposition nor the genetic structure of bacterial communities in response to changes in N availability, but displayed higher specific N uptake and total N content in response to increased N availability. As a result, the increase of specific N uptake under high N availability was probably not due to the presence of a specific microbial community but to a better efficiency of the autonomous pathway for N acquisition. According to Moreau et al. [25], the N uptake of *Medicago truncatula* cv. Jemalong line A17 was not able to match the plant N requirements when symbiotic N_2_ fixation was the main source of N. Thus for this group of genotypes, the carbon cost invested in plant-microbe interactions was lower when N availability increased. It is also probable that the mineral N uptake by plant is more efficient than symbiotic N_2_ fixation.

Therefore, plant-microbe interactions differentially responded to N availability according to the *Medicago truncatula* genotype through structural and functional modifications of the plant traits involved in the interaction.

N availability effect on bacterial communities could be plant-mediated through changes in carbon fluxes from plant to soil, as rhizosphere bacterial communities are considered as the most important decomposers of exudates [61]. According to Liljeroth et al. [62], rhizodeposition, microbial biomass and rhizosphere bacterial numbers increase in response to a higher N availability in the soil. Indeed, the N status of the plant could affect root exudation and microbial colonisation [26]. In our study, a significant effect of N availability was observed for the genetic structure of bacterial communities of group 1 and 2. Contrastingly, if a higher rhizodeposition was observed for group 2 under high N availability, it was not the case of group 1. However, even if no quantitative difference in rhizodeposition was observed for group 1 in response to a modification of N availability, it cannot be excluded that qualitative differences in rhizodeposition could be at the origin of the variations of group 1 associated bacterial communities. Indeed, Micallef et al. [63] suggested that a difference in exudation patterns among *Arabidopsis thaliana* genotypes could influence bacterial assemblage.

In conclusion, our study demonstrated that *Medicago truncatula* genotypes and their adaptive strategies to environmental constraints (here soil N availability), are major components in the shaping of rhizosphere microbial communities. In the future, a new challenge will be to focus on the roles played by the plant genetic and environmental determinisms on the outcome of the plant-microbial community interaction. With this new understanding of these interactions, new ideotypes showing enhanced root interactions with beneficial microorganisms and nutrient acquisition efficiency could thus be designed to improve agricultural performances.

## Supporting Information

Table S1 **Level of symbiotic association for different genotypes of** ***Medicago truncatula*** **and N treatments.** ^a^Intensity of nodulation was visually assessed at 934 degree-days after sowing, using a qualitative scale (Moreau et al. 2008). The scale includes five scores: 0 (absence of nodules), 1 (only some white nodules of small size), 2 (both white and pink nodules of small size), 3 (mainly pink nodules of larger size), and 4 (many pink nodules of large size). ^b^Mycorrhization parameters represented the frequency (F%) and intensity of colonisation of the root cortex (M%) and the arbuscular abundance of the mycorrhized root cortex (A%) at 934 degree-days after sowing.
(DOCX)

Table S2 **Developmental characterization and C and N nutritional strategies of different** ***Medicago truncatula*** **genotypes under two contrasted N treatments (0 and 10 mM N).** SNU represents the specific nitrogen uptake. SNU is the correlation coefficient of the function between the total amount of nitrogen (g plant^−1^) and below-ground biomass (g plant^−1^). NLA represents the conversion factor of nitrogen to leaf area. NLA is the correlation coefficient of the function between leaf area (cm^2^ plant^−1^) and the total amount of nitrogen (g plant^−1^). RUE represents the radiation use efficiency. RUE is the correlation coefficient of the function between total biomass (g plant^−1^) and the sum of intercepted PAR (MJ plant^−1^). Root∶total biomass ratio is the correlation coefficient of the function between below-ground biomass (g plant^−1^) and total biomass (g plant^−1^). SNU, NLA, RUE and root∶total ratio were calculated from three dates (340, 634 and 934 degree-days) and 4 plant repetitions.
(DOCX)
