# Supplementary material for: Soil Nitrogen Availability and Plant Genotype Modify the Nutrition Strategies of M. truncatula and the Associated Rhizosphere Microbial Communities
Source: PLoS One. 2012 Oct 15;7(10):e47096. doi: 10.1371/journal.pone.0047096 (PMC3471967; doi:10.1371/journal.pone.0047096)
Supplement: Table S1 — Level of symbiotic association for different genotypes of Medicago truncatula and N treatments. aIntensity of nodulation was visually assessed at 934 degree-days after sowing, using a qualitative scale (Moreau et al. 2008). The scale includes five scores: 0 (absence of nodules), 1 (only some white nodules of small size), 2 (both white and pink nodules of small size), 3 (mainly pink nodules of larger size), and 4 (many pink nodules of large size). bMycorrhization parameters represented the frequency (F%) and intensity of colonisation of the root cortex (M%) and the arbuscular abundance of the mycorrhized root cortex (A%) at 934 degree-days after sowing. (DOCX) [file pone.0047096.s001.docx]

**Table S1.** **Level of symbiotic association for different genotypes of *Medicago truncatula* and N treatments.**

| **Genotype** | **Treatment (mM N)** | **Intensity of nodulation ^a^** | **Mycorrhization parameters ^b^** | | |
| --- | --- | --- | --- | --- | --- |
|  |  |  | **F%** | **M%** | **A%** |
| DZA315-16 | 0 | 4.0 | 92.7 | 35.2 | 29.8 |
|  | 10 | 1.0 | 87.3 | 48.2 | 37.0 |
| DZA315-26 | 0 | 4.0 | 89.3 | 32.7 | 29.5 |
|  | 10 | 0.8 | 91.3 | 49.5 | 38.1 |
| F83005-5 | 0 | 4.0 | 95.3 | 44.8 | 38.9 |
|  | 10 | 0.5 | 88.7 | 47.0 | 35.0 |
| SA028064 | 0 | 3.3 | 90.8 | 42.6 | 39.7 |
|  | 10 | 0.8 | 92.7 | 46.3 | 39.6 |
| Jemalong-A17 | 0 | 3.5 | 86.7 | 36.2 | 29.5 |
|  | 10 | 0.3 | 90.7 | 43.0 | 34.7 |
| Jemalong-J6 | 0 | 3.8 | 90.7 | 43.3 | 36.6 |
|  | 10 | 0.8 | 90.0 | 41.1 | 31.0 |

^a^ Intensity of nodulation was visually assessed at 934 degree-days after sowing, using a qualitative scale (Moreau et al. 2008). The scale includes five scores: 0 (absence of nodules), 1 (only some white nodules of small size), 2 (both white and pink nodules of small size), 3 (mainly pink nodules of larger size), and 4 (many pink nodules of large size).

^b^ Mycorrhization parameters represented frequency (F%) and intensity of colonization of the root cortex (M%) and arbuscular abundance of the mycorrhized root cortex (A%) at 934 degree-days after sowing.
