# Supplementary material for: Soil Nitrogen Availability and Plant Genotype Modify the Nutrition Strategies of M. truncatula and the Associated Rhizosphere Microbial Communities
Source: PLoS One. 2012 Oct 15;7(10):e47096. doi: 10.1371/journal.pone.0047096 (PMC3471967; doi:10.1371/journal.pone.0047096)
Supplement: Table S2 — Developmental characterization and C and N nutritional strategies of different Medicago truncatula genotypes under two contrasted N treatments (0 and 10 mM N). SNU represents the specific nitrogen uptake. SNU is the correlation coefficient of the function between the total amount of nitrogen (g plant−1) and below-ground biomass (g plant−1). NLA represents the conversion factor of nitrogen to leaf area. NLA is the correlation coefficient of the function between leaf area (cm2 plant−1) and the total amount of nitrogen (g plant−1). RUE represents the radiation use efficiency. RUE is the correlation coefficient of the function between total biomass (g plant−1) and the sum of intercepted PAR (MJ plant−1). Root∶total biomass ratio is the correlation coefficient of the function between below-ground biomass (g plant−1) and total biomass (g plant−1). SNU, NLA, RUE and root∶total ratio were calculated from three dates (340, 634 and 934 degree-days) and 4 plant repetitions. (DOCX) [file pone.0047096.s002.docx]

**Table S2. Developmental characterization and C and N nutritional strategies of different *Medicago truncatula* genotypes under two contrasted N treatments (0 and 10 mM N).**

| **Genotype** | **Treatment**  **(mM N)** | **Leaf area (cm²)** | **Total biomass (g)** | **Root:total biomass ratio** | **RUE (g of biomass MJ^-1^ of PAR intercepted)** | **Rhizodeposition (µ C s^-1^ g^-1^ of root)** | **NNI** | **Total amount of N (mg.plant^-1^)** | **SNU (g of N g^-1^ of root biomass)** | **NLA (cm² of leaves g^-1^ of N)** |
| --- | --- | --- | --- | --- | --- | --- | --- | --- | --- | --- |
| DZA 315-16 | 0 | 111.6 | 1.31 | 0.28 | 3.53 | 0.022 | 0.48 | 33 | 85.7 | 3186 |
|  | 10 | 99.1 | 1.09 | 0.33 | 3.04 | 0.032 | 0.70 | 37 | 94.6 | 2514 |
| DZA 315-26 | 0 | 83.6 | 0.92 | 0.33 | 3.28 | 0.024 | 0.47 | 24 | 78.4 | 3137 |
|  | 10 | 72.6 | 0.73 | 0.39 | 4.49 | 0.035 | 0.67 | 26 | 90.6 | 2681 |
| F 83005-5 | 0 | 94.7 | 1.13 | 0.30 | 3.38 | 0.018 | 0.49 | 30 | 79.0 | 2990 |
|  | 10 | 96.4 | 1.09 | 0.33 | 2.98 | 0.028 | 0.69 | 40 | 111.4 | 2332 |
| SA 028064 | 0 | 76.4 | 1.07 | 0.28 | 4.04 | 0.011 | 0.41 | 25 | 78.0 | 3004 |
|  | 10 | 98.1 | 1.09 | 0.31 | 3.35 | 0.024 | 0.61 | 37 | 105.5 | 2560 |
| Jemalong A17 | 0 | 88.6 | 1.28 | 0.34 | 4.73 | 0.020 | 0.45 | 31 | 69.5 | 2690 |
|  | 10 | 92.6 | 1.21 | 0.34 | 4.29 | 0.027 | 0.68 | 43 | 102.2 | 2074 |
| Jemalong J6 | 0 | 77.9 | 1.08 | 0.35 | 4.12 | 0.021 | 0.42 | 24 | 60.8 | 2973 |
|  | 10 | 84.3 | 1.15 | 0.28 | 4.84 | 0.028 | 0.67 | 39 | 109.3 | 2028 |

SNU represents the specific nitrogen uptake. SNU is the correlation coefficient of the function between the total amount of nitrogen (g plant^-1^) and below-ground biomass (g plant^-1^). NLA represents the conversion factor of nitrogen to leaf area. NLA is the correlation coefficient of the function between leaf area (cm² plant^-1^) and the total amount of nitrogen (g plant^-1^). RUE represents the radiation use efficiency. RUE is the correlation coefficient of the function between total biomass (g plant^-1^) and the sum of intercepted PAR (MJ plant^-1^). Root:total biomass ratio is the correlation coefficient of the function between below-ground biomass (g plant^-1^) and total biomass (g plant^-1^). SNU, NLA, RUE and root:total ratio were calculated from three dates (340, 634 and 934 degree-days) and 4 plant repetitions.
